# Modified Lund concept versus cerebral perfusion pressure-targeted therapy: a randomized controlled study in patients with secondary brain ischaemia

**DOI:** 10.1186/cc9762

**Published:** 2011-03-11

**Authors:** MA Hamdan, K Dizdarevic

**Affiliations:** 1 Newcastle University, Newcastle upon Tyne, UK; 2 Clinical Centre University of Sarajevo, Sarajevo, Bosnia and Herzegovina

## Introduction

Secondary brain ischaemia (SBI) usually develops after aneurysmal subarachnoid haemorrhage (SAH) and severe traumatic brain injury (TBI). The current management strategies are based on intracranial pressure-targeted therapy (ICP-targeted) with cerebral microdialysis monitoring (modified Lund concept) or cerebral perfusion pressure-targeted therapy (CPP-targeted) [1-3]. We present a randomised controlled study to compare the two management strategies.

## Methods

Sixty comatose operated patients with SBI following aneurysmal SAH and severe TBI were randomized into ICP-targeted therapy with cerebral microdialysis monitoring and CPP-targeted therapy groups. Mortality rates in both groups were calculated and biochemical signs of cerebral ischaemia were analysed using cerebral microdialysis. Outcome for cerebral microdialysis was measured as poor outcome (Glasgow Outcome Scale score 1, 2 and 3) or good outcome (Glasgow Outcome Scale score 4 and 5).

## Results

Patients treated by ICP-targeted therapy with cerebral microdialysis monitoring had a significantly lower mortality rate compared with those treated by CPP-targeted therapy (*P *= 0.03). Patients undergoing cerebral microdialysis with poor outcome had lower mean values of glucose and higher mean values of glycerol and lactate/pyruvate ratio as compared with those with good outcome (glucose: *P *= 0.003; glycerol: *P *= 0.02; lactate/pyruvate ratio: *P *= 0.01). There was no difference in the outcome between aneurysmal SAH and severe TBI in the two groups.

## Conclusions

The ICP-targeted therapy based on modified Lund concept showed better results compared with CPP-targeted therapy in the treatment of comatose patients sustaining SBI after aneurysmal SAH and severe TBI.
